# What Qualities Are Most Important to Making a Point of Care Test Desirable for Clinicians and Others Offering Sexually Transmitted Infection Testing?

**DOI:** 10.1371/journal.pone.0019263

**Published:** 2011-04-29

**Authors:** Yu-Hsiang Hsieh, Charlotte A. Gaydos, M. Terry Hogan, O. Manuel Uy, Joany Jackman, Mary Jett-Goheen, Ariel Albertie, Derek T. Dangerfield, Celia R. Neustadt, Zachary S. Wiener, Anne M. Rompalo

**Affiliations:** 1 Department of Emergency Medicine, Johns Hopkins University School of Medicine, Baltimore, Maryland, United States of America; 2 Division of Infectious Diseases, Johns Hopkins University School of Medicine, Baltimore, Maryland, United States of America; 3 Milton Eisenhower Research Center, Johns Hopkins University Applied Physics Laboratory, Laurel, Maryland, United States of America; University of Cape Town, South Africa

## Abstract

**Background:**

To investigate the possible effects of different levels of attributes of a point-of-care test (POCT) on sexually transmitted infection (STI) professionals' decisions regarding an ideal POCT for STI(s).

**Methods:**

An online survey was designed based on a large-scale in-depth focus discussion study among STI experts and professionals. The last section of the survey “build your own POCT” was designed by employing the discrete choice experiment approach. Practicing clinicians from two venues, STI-related international conference attendees and U.S. STD clinic clinicians were invited to participate in the survey. Conditional logistical regression modeling was used for data analysis.

**Results:**

Overall, 256 subjects took the online survey with 218 (85%) completing it. Most of the participants were STD clinic clinicians who already used some POCTs in their practice. “The time frame required” was identified as a major barrier that currently made it difficult to use STI POCTs. *Chlamydia trachomatis* was the organism chosen as the top priority for a new POCT, followed by a test that would diagnose early seroconversion for HIV, and a syphilis POCT. Without regard to organism type selected, sensitivity of 90–99% was always the most important attribute to be considered, followed by a cost of $20. However, when the test platform was prioritized for early HIV seroconversion or syphilis, sensitivity was still ranked as most important, but specificity was rated second most important.

**Conclusions:**

STI professionals preferred *C. trachomatis* as the top priority for a new POCT with sensitivity over 90%, low cost, and a very short completion time.

## Introduction

Sexually transmitted infections (STIs), the leading group of reportable diseases in the United States each year, have an estimate of approximately 20 million new cases [1] and more than 10 billion dollars in costs each year [2]. A good point-of-care test (POCT) for STI(s) which may offer immediate diagnosis and prompt treatment could effectively reduce prevalence and transmission in communities. World Health Organization Sexually Transmitted Diseases Diagnostics Initiative (WHO SDI) has identified the following test criteria as benchmarks for a POCT for STI(s) which would have the ability to address some of STI control needs: Affordable, Sensitive, Specific, User-friendly, Rapid and robust, Equipment-free and Deliverable to end-users (ASSURED) [3], [4].

A recent large-scale in-depth focus group discussion study among STI experts and professionals showed that high sensitivity and specificity, quick turn-around time, and low cost were the most important characteristics for an ideal POCT for STIs [5]. However, in reality, a diagnostic test may not possess all of these ideal attributes that everyone desires. Little is known about which characteristics STI professionals value over others when they are forced to choose between different diagnostic characteristics that have significant advantages and disadvantages. Choice questions that vary attributes can measure how STI professionals would ‘trade off’ different level of sensitivity, specificity, cost, and time for a test, i.e. prefer one test over another another test, each having a defined set set of values and characteristics.

The discrete-choice experiment was originally developed in marketing research in the early 1970s as an attribute-based measure methodology which gauges individual's evaluation on levels of attributes/characteristics of a service, policy, intervention, or a diagnostic test for decision-making [6], [7]. This approach is increasingly applied in health care and health economics research [8], [9], [10]. Our goal was to investigate the possible effects of different levels of attributes of POCT, including sensitivity, specificity, turn-around time, and cost, on STI professionals' decisions regarding an ideal POCT for STI(s) by employing the discrete-choice experimental approach.

## Materials and Methods

### Ethical Statement

This study protocol was approved by Institutional Review Board (IRB) of The Johns Hopkins University School of Medicine. Due to the nature of our online survey study design as well as the research presented no more than minimal risk to subjects, verbal or conventional written consent was not obtained per The Johns Hopkins University School of Medicine IRB approval. We also included a written disclosure describing the study in the beginning of the survey. Subjects were able to terminate the survey at any time.

### Study Population/Recruitment

A list of all categorical STI clinics throughout the ten federally funded regions of the United States was provided by the Centers for Disease Control and Prevention. The list was validated by research assistants who divided the list according to region and telephoned each designated listed clinic manager within the assigned regions to invite them to participate in the online survey. Each manager was asked to distribute an invitation letter to all of their clinicians to also participate in the online survey (in the hope that clinicians would be more likely to take the survey if it were recommended by a co-worker). An IRB-approved invitation letter and a thank you e-mail were then sent to each agreeable clinic manager. We were able to contact over 700 clinics offering STI care and collected information from over 200 respondents. In addition to the U.S. based participants, we further recruited survey participants via in-person outreach among attendees at two international conferences: the International Society for Sexually Transmitted Diseases Research (ISSTDR) conference held in London (June 2009) and the Infectious Diseases Society for Obstetrics and Gynecology in Montreal (August 2009).

### Survey Tool

The survey (Appendix S1) was developed based on the findings from our formative assessment project which identified the need for and perception of qualities imperative for an ideal new STI POCT [5]. Our survey contained the following elements: (1) demographics, including gender, country of practice, profession, type of practice; (2) currently available POCTs and unique barriers of use; (3) ideal future type of POCT for STI(s), including prioritizing the pathogens for development and economic factors; and (4) a section called “build your own POCT” - preference of POCT for STI(s) with different levels of sensitivity, specificity, turn-around time, and cost. The last part of the survey was designed using the discrete choice experiment approach. We used 3 levels of sensitivity (70–79%, 80–89%, ≥90%), 3 levels of specificity (90%, 95%, 99%), 3 levels of turn-around time (5 minutes, 15 minutes, 25 minutes), and 3 levels of cost ($20, $35, $50) to randomly create 16 choice questions. Each of the choice questions contained a pair of POCTs with different sets of attributes for participants to select their preferred diagnostic from the pair. The survey was placed on Survey Monkey (SurveyMonkey.com, Portland, OR) after several rounds of pre-tests of the survey tool.

### Data Analysis

Data were described using frequencies and percentages as appropriate. In the “build your own POCT” section of survey data, the probability of individuals making a particular choice from presented alternatives were estimated by choice modeling which is a type of conditional logistic regression. Odds ratios were calculated from regression coefficients for all attributes. Subgroup analyses of choice modeling were performed for each of top three prioritized pathogens for new STI POCT chosen by the participants, as well as profession (medical director versus and non medical director) and geographical region (U.S. versus non-U.S.). SAS version 9.2 and JMP version 8 (SAS Institute Inc., Cary, NC) were used for analysis purposes. All p values were 2-sided, with p<0.05 considered to be significant.

## Results

Overall, 256 subjects took the online survey and 218 (85%) completed the survey. There were no statistical differences in gender, country of residency, and profession between subjects who completed the survey and those who did not. Seventy-nine (36%) were conference participants [ISSTDR: 52 (24%); IDSOG: 27 (12%)]. One hundred thirty-nine (64%) of participants were STD clinic clinicians. Demographic characteristics of 218 respondents are presented in Table 1.

**Table 1:** Demographic Characteristics of 218 Respondents who Participated in the Survey: “Build Your Own” Point-of-Care Test.

Characteristics	Categories	Number (%)
		N = 218
Source of Participants	ISSTDR	52 (23.8)
	IDSOG	27 (12.4)
	STD Clinics	139 (63.8)
Gender	Male	48 (22.0)
	Female	170 (78.0)
Country or Continent of Residence	America	
	United States	169 (77.5)
	Canada	3 (1.2)
	Other	3 (1.2)
	Europe	
	United Kingdom	16 (7.3)
	Other	7 (3.2)
	Asia	6 (2.8)
	Africa	5 (2.2)
	Oceania	2 (0.9)
	Unknown	7 (3.2)
Profession	Registered Nurse	85 (39.0)
	Medical Director	67 (30.7)
	Nurse Practitioner	22 (10.1)
	Clinical Manager	11 (5.0)
	Laboratory Director	8 (3.7)
	Laboratory Technician	5 (2.2)
	Other	20 (9.2)
Location of Practices	Inner City	80 (36.7)
	Rural	56 (25.7)
	Non-inner City Urban	54 (24.8)
	Suburban	28 (12.8)
Primary Practice	Public Health Clinic	164 (75.2)
Medicaid/Medicare Provider	Yes	93 (42.7)

The most commonly available STI POCT at participants' clinics currently was the wet mount preparation test for the microscopic detection of trichomonas, yeast, and “clue cells” (78%), i.e. saline and/or potassium hydroxide (KOH) slide preparations of vaginal fluid for trichomonas, yeast and bacterial vaginosis diagnosis respectively. Urine dipstick (70%) was the second most mentioned assay, followed by the rapid HIV test (60%), vaginal pH determination (56%), Gram Stain (48%), and rapid syphilis test, i.e. rapid plasma reagin (RPR) (20%).

Among a list of barriers, 40% of participants identified that ‘the time frame required’ was the most significant barrier that would make it difficult to use currently available STI POCTs. Four other barriers, including complexity with multiple steps (31%), interruption of work flow (30%), perceived wait time for patients (30%), laboratory driven (30%), were reported by similar proportions of participants. A considerable number of participants pointed out that ‘unreliability’ (23%) and ‘time-step driven’ (too many timed steps in performance of the test) characteristics (16%) of POCT would make it harder to use in the clinic. Fewer indicated that ‘difficulty in reading results (10%), ‘cost’ (8%), and ‘invasiveness’ (7%) were barriers. When being asked to select one barrier that would make it hardest for them to use STI POCT, participants had different opinions. Several barriers emerged as leading choices that would make it hardest to use, including ‘laboratory driven’ (17%), ‘time frame’ (14%), ‘complexity’ (12%), ‘unreliability’ (12%), ‘interruption of work flow’ (11%), and ‘perceived wait time for patients’ (10%).

When participants were asked to rank their top three choices for organisms in need of a POCT for STIs, *Chlamydia trachomatis* was significantly ranked as the top priority for development of new POCT by 62% of participants (p<0.05), followed by detection of early seroconversion of HIV infection (14%), and a POCT for syphilis (8%). There was no statistical difference in the number of subjects who chose HIV seroconversion and the number of those who chose syphilis. As a second priority test, 35% of participants chose gonorrhea followed by chlamydia (15%), HBV and/or HCV (10%), and syphilis (10%). Gonorrhea (22%) was the leading choice as third priority, followed by herpes simplex virus (19%), and syphilis (13%).

The majority (78%) of participants believed that the cost of the test from the manufacturer was an important factor in designing a POCT, while less a quarter (22%) of participants believed the amount of reimbursement received for performing the test to be vital. Medical directors did not have different concerns in this issue as compared to those who were not medical directors (p = 0.38).

### Build Your Own Test

Generally speaking, a test with a high level of sensitivity (90%) was overwhelmingly preferred over the one with a low level of sensitivity (70%). A test with a low level of cost ($20) was also preferred over the one with a high level of cost ($50). Some highlights of comparison of the individual choice question sets were as follows. A lower level of sensitivity (70%) of POCT was preferred over a medium level of sensitivity (80%) if it had low cost ($20) and fast turn-over-time (5 minutes) (Appendix S1 Section “Build your own test” – Question 3) or if it had a high level of specificity (99%) and low cost (Appendix S1 Section “Build your own test” – Question 4). The majority also thought that a high level of sensitivity (90%) could be traded for a medium level of sensitivity if the POCT had a higher specificity and was much cheaper (Appendix S1 Section “Build your own test” – Question 5). High specificity was more important over lower specificity even if the test was in a category higher in cost and a category slower in time (Appendix S1 Section “Build your own test” – Question 14).

Using choice modeling, we found that all participants who completed the survey selected sensitivity as their top priority issue for a building a new STI POCT, followed by cost, specificity, and time. They preferred the new POCT to have a sensitivity of 90–99%, a cost of $20, a specificity of 99%, and a turn-around-time of 5 minutes (Table 2). Further subgroup analyses on top three priorities for new POCTs based on a specific individual disease demonstrated some differences in the perceived preference in these 4 attributes. Participants still ranked sensitivity as the leading consideration; however, specificity became the second most important factor for those who chose HIV seroconversion or syphilis as their priority for a new POCT, rather than cost which was chosen by those preferred *C. trachomatis* as the priority assay.

**Table 2:** Regression Analysis of the Importance of Preferences in Attributes of a New-Point-of-Care Test for Sexually Transmitted Infections by All Tests and by Prioritized Tests.

Attributes		Odds Ratios			
		ALLN = 218	ChlamydiaN = 136	Early HIV SeroconversionN = 30	SyphilisN = 21
Sensitivity (%)	90–99	13.6*	18.2*	10.6*	11.8*
	80–90	4.1*	4.7*	3.1*	4.6*
	70–80	1.0	1.0	1.0	1.0
Specificity (%)	99	3.7*	3.7*	4.7*	5.9*
	95	2.2*	2.1*	2.4*	3.1*
	90	1.0	1.0	1.0	1.0
Cost ($)	20	4.5*	5.2*	3.2*	4.3*
	35	2.1*	2.3*	1.8*	2.1*
	50	1.0	1.0	1.0	1.0
Time (minutes)	5	3.0*	3.2*	2.5*	3.6*
	15	1.7*	1.8*	1.6*	1.9*
	25	1.0	1.0	1.0	1.0

* p<0.05.

In subgroup analyses of professional differences or on geographical regions, sensitivity was still reported as the top priority and time was the least priority for all subgroups. However, specificity was the second most important priority for those who were medical directors, while cost was the second one for those who were not medical directors (data not shown). In addition, subjects from U.S. preferred cost as second priority issue over specificity while cost and specificity were tied for the second priority issue (data not shown).

## Discussion

Our survey study gathered opinions on an ideal POCT for STIs from a large group of practicing clinicians who use and would use new POCTs for STIs. It continued the progression path to the second phase of needs assessment for an ideal POCT for STIs following our large-scale in-depth focus discussion study among STI experts and professionals. This larger more extensive quantitative survey further confirmed our findings from the qualitative focus group discussion study [5]. In addition, it contained choice questions to understand which characteristics STI professionals value over others when they are forced to choose between different diagnostic characteristics that have significant advantages and disadvantages. Without regard to type of assay selected (i.e. all POCTs considered together without regard to organism type), sensitivity of 90–99% was always the most important attribute to be considered. Our participants clearly stated what they most desired for a POCT, which is important for manufacturers, as well as public health officials and regulatory organizations to consider in order to avoid developing and approving low sensitivity POCTs as van Dommelen *et al.* found recently [11]. The second most important characteristic named by participants was cost when all POCTs were considered in aggregate, with costs or $20 or less being consistently named in regression analysis containing all characteristics. It was surprising that cost was such a large factor in the consideration of attributes surveyed.

Chlamydia was the organism chosen most often as the top priority for a new POCT. When the choice question analysis was stratified by the particular type of organism chosen, the odds ratios changed somewhat as to importance of attribute from when all types of tests were aggregated together and are probably more important to consider in specific test development. The difference in priority ranking order might be due to the differential impact of false positive result by pathogen on the subjects who receive POCT. Due to the nature of our survey study design, we were unable to further determine the reasons, e.g. why specificity was ranked lower than cost by our participants who chose chlamydia as the top priority pathogen. Further future studies are warranted to elucidate this issue.

“Choice experiment” preference is a type of survey that presents the potential user and/or buyer with a choice among several prospective product offerings. This type of experiment can help researchers, manufacturers, and retailers identify the most important product attributes and assign parameter importance values to them. By forcing choices as to combinations of different levels of sensitivity, specificity, turn-around time, and cost to randomly create “choice questions”, regression analysis was able to discern which trade-offs of choices were most important to the participants, by using only 16 choice questions in the survey. Multiple parameters and attributes were varied in each choice question in order to ascertain which were most important to the potential user and which characteristics were “negotiable” in order to build an ideal test, recognizing that not all perfect attributes were achievable. Participants were forced to make choices in order to preserve what were the most desirable features of a POCT.

Sensitivity was still the top priority for a building a new STI POCT among 4 attributes that we investigated in this study for participants in terms of their profession and country of residence. However, second priority was different by profession and country of residence. In general, medical directors in our survey might be more concerned with false positive issue than financial issues, resulting in their preference in specificity over cost. For those who were not medical directors, they were more concerned with financial issues. Reimbursement might be a more relevant issue with testing in U.S. Therefore, participants from U.S. preferred cost as second priority issue over specificity while the rest of world viewed cost and specificity as evenly important.

Many barriers to routinely using POCTs for STIs were identified by survey participants, including that ‘the time frame required’ was the barrier that would make it difficult to use currently available STI POCTs. Many other barriers to use were commented on, such as the complexity with multiple steps required, interruption of clinic work flow, perceived wait time for patients, and requirements that the test be performed in a laboratory rather than at the clinic office (laboratory driven). All of these considerations could be prohibitive and could potentially be “show stoppers” for adoption of a new POCT, unless thought is given to these potential problems when tests are designed. The ranking of desired attributes of a useful POCT for STIs could assist assay developers to design tests that meet the ASSURRED characteristics, such that new tests will fulfill the usability criteria [3], [4].

Our study population, attendees of two STI-related international conferences who were recruited face-to-face and U.S. STD clinic clinicians who were recruited by phone calls and referral from their colleagues, might be not representative to all STI clinicians and professionals. Therefore, generalizability of our findings could be an issue and are a limitation of this type of approach. Nevertheless, our participants were a wide range of types of clinicians actively working in STD clinics in different types of practice settings from inner city to rural. More importantly, they have already used some POCTs in their practice, making the opinions and preferences collected from our survey study from these current STI ‘POCT’ users imperative for the development of new POCT for STI(s). Another potential limitation of this study is that the potential possibility of “building your own test” appears to be skewed to all “positive” attributes which will provide a high degree of bias to select the highest sensitivity and specificity at the lowest cost and shortest time, which might be technically infeasible. However, the high degree of bias is likely avoided since we randomly created our 16 choice set questions for “building your own test” in which participants had to select one hypothetically “imperfect” POCT over another “imperfect” one in all but one choice sets. Finally, most of the participants were clinical providers offering STI testing to their patients in the frontline. They might not know that the “ideal” test, i.e. highest level of sensitivity and specificity at the lowest cost and shortest time in the choice questions, is not likely technically feasible currently. However, their responses provide industry as well as academia what they desired for in POCT STI when offering STI testings for their clients. A POCT STI with these preferred ideal characteristics might become a reality if the technology has some breakthrough in the near future.

In summary, our study provided pilot information identifying the need for and the preference for a set of certain attributes among several options with different level of attributes for an ideal new STI POCT from STI professional end users. Our findings serve as some of latest guidance and direction of the development of a new and ideal STI POCT for use by practitioners working in public health, academia, and industry. Such information may be valuable in avoiding to design a test which gives unsatisfactory results for POC testing results [12].

## Supporting Information

Appendix S1 Point-of-Care Testing Online Survey.
(PDF)
