# Supplementary material for: What Qualities Are Most Important to Making a Point of Care Test Desirable for Clinicians and Others Offering Sexually Transmitted Infection Testing?
Source: PLoS One. 2011 Apr 29;6(4):e19263. doi: 10.1371/journal.pone.0019263 (PMC3084795; doi:10.1371/journal.pone.0019263)
Supplement: Appendix S1 — Point-of-Care Testing Online Survey. (PDF) [file pone.0019263.s001.pdf]

Point-of-Care Testing Survey

Welcome

Thank you for taking the time to perform our survey. The purpose of this study is to determine what qualities are most important to making a Point of Care Test desirable for clinicians and others offering STI testing. Our hope is that the results of this study will be used to design an ultimate Point of Care Test that is effective and efficient.

The survey should take approximately 10 minutes.

Before beginning the survey, we'd like to ask you a few questions about who referred you to this study. After answering these questions, please choose the "next" button to begin the survey.

How did you hear about this survey?

What is your referral number?
(This can be found in the email that was sent to you, which contained the link to this survey)

Point-of-Care Testing Survey

Practice Demographics

Please tell us a little bit about yourself and your practice.
To answer the following questions, please select a response from the drop down menus.

What is your gender?

[ ]

In which country do you practice primarily?

[ ]

What is your profession?

- [ ] MD - Medical Doctor
- [ ] DO - Doctor of Osteopathy
- [ ] NP - Nurse Practitioner
- [ ] CNM - Certified Nurse Midwife
- [ ] APN - Advanced Practice Nurse
- [ ] PA - Physician's Assistant
- [ ] RN - Registered Nurse
- [ ] Clinic Manager
- [ ] Laboratory Director
- [ ] Laboratory Technician
- [ ] Health Aide
- [ ] Other

Point-of-Care Testing Survey

If you answered "Other" to the question above, please elaborate briefly on your work as it pertains to STD diagnostics.
To answer this question, please type a response in the box viewed directly below.

Where is your practice located?

What is the best approximation of your primary practice?

- [ ] Public Health Clinic
- [ ] Federally Qualified Health Center
- [ ] Private - Single Practitioner Setting
- [ ] Private - Multiple Practitioner Setting
- [ ] Private Community Clinic
- [ ] Health Maintenance Organization
- [ ] College/University

Are you a Medicaid / Medicare managed care provider?

Point-of-Care Testing Survey

Current Access

Point of Care Tests for Sexually Transmitted Infections are defined as tests that can be done onsite with results available during the clinic visit and before the patient leaves.

Please indicate which Point-of-Care tests are available to you currently (mark all that apply)

- ☐ Vaginal pH
- ☐ Gram stain
- ☐ Stat RPR
- ☐ Wet-Mount test (saline and potassium hydroxide)
- ☐ Urine dipstick
- ☐ Rapid HIV test (any kind)
- ☐ Pregnancy test
- ☐ Other

Other (please specify)

Point-of-Care Testing Survey

Which do you identify as barriers that make it harder to use Point-of-Care Testing?

- [ ] Complexity (multiple steps)
- [ ] Time frame
- [ ] Time-step driven
- [ ] Laboratory driven
- [ ] Difficulty reading results
- [ ] Interruption of workflow
- [ ] Unreliability
- [ ] Invasiveness
- [ ] Perceived wait times for patients
- [ ] Other

Other (please specify below)

Point-of-Care Testing Survey

Of these barriers, which one makes it hardest for you to use Point-Of-Care testing?

- ◯ Complexity (multiple steps)
- ◯ Time frame
- ◯ Time-step driven
- ◯ Laboratory driven
- ◯ Difficulty reading results
- ◯ Interruption of workflow
- ◯ Unreliability
- ◯ Invasiveness
- ◯ Perceived wait times for patients
- ◯ Other

Other (please specify below)

Future tests

If a Point of Care Test were to be developed for one of these STI conditions, which condition would you choose as your FIRST priority for development?

- ◯ Chlamydia
- ◯ Gonorrhea
- ◯ Hepatitis B and/or C
- ◯ Herpes Simplex Virus
- ◯ HIV (early seroconversion)
- ◯ Human papillomavirus
- ◯ Syphilis
- ◯ Trichomonas

Which condition would you choose as your SECOND priority for development?

- ◯ Chlamydia
- ◯ Gonorrhea
- ◯ Hepatitis B and/or C
- ◯ Herpes Simplex Virus
- ◯ HIV (early seroconversion)
- ◯ Human papillomavirus
- ◯ Syphilis
- ◯ Trichomonas

Point-of-Care Testing Survey

Which condition would you choose as your THIRD priority for development?

- ◯ Chlamydia
- ◯ Gonorrhea
- ◯ Hepatitis B and/or C
- ◯ Herpes Simplex Virus
- ◯ HIV (early seroconversion)
- ◯ Human papillomavirus
- ◯ Syphilis
- ◯ Trichomonas

Which economic factor is more important to you in designing a test?

- ◯ The cost of the test from the manufacturer
- ◯ The amount of reimbursement received for performing the test

Build your own test - Question 1

The next 16 questions are designed to determine what Point of Care Test is most important to you. Answer the following questions with your highest priority STI in mind. For example, if you chose chlamydia as your priority STI for Point of Care Testing, then you should answer the questions as if the Point of Care Test you are designing is for chlamydia.

The table shows the probabilities of a correct diagnosis in the presence of the disease, a correct diagnosis in the absence of the disease, the cost of the test, and the time to complete (in minutes).

	Probability of Correctly Diagnosed Positive	Probability of Correctly Diagnosed Negative	Cost Per Test	Time to Complete
Test 1	80 – 90%	90%	$35	5 min.
Test 2	70 – 80%	95%	$35	25 min.

Which test better suits your needs?

- ◯ Test 1
- ◯ Test 2

Build your own test - Question 2

	Probability of Correctly Diagnosed Positive	Probability of Correctly Diagnosed Negative	Cost Per Test	Time to Complete
Test 3	80 – 90%	90%	$35	25 min.
Test 4	80 – 90%	99%	$20	15 min.

Which test better suits your needs?

◯ Test 3

◯ Test 4

Point-of-Care Testing Survey

Build your own test - Question 3

	Probability of Correctly Diagnosed Positive	Probability of Correctly Diagnosed Negative	Cost Per Test	Time to Complete
Test 5	70 – 80%	95%	$20	5 min.
Test 6	80 – 90%	95%	$50	15 min.

Which test better suits your needs?

- ◯ Test 5
- ◯ Test 6

Point-of-Care Testing Survey

Build your own test - Question 4

	Probability of Correctly Diagnosed Positive	Probability of Correctly Diagnosed Negative	Cost Per Test	Time to Complete
Test 7	80 – 90%	95%	$50	25 min.
Test 8	70 – 80%	99%	$20	25 min.

Which test better suits your needs?

- ◯ Test 7
- ◯ Test 8

Point-of-Care Testing Survey

Build your own test - Question 5

	Probability of Correctly Diagnosed Positive	Probability of Correctly Diagnosed Negative	Cost Per Test	Time to Complete
Test 9	90 – 99%	90%	$50	25 min.
Test 10	80 – 90%	95%	$20	25 min.

Which test better suits your needs?

- ◯ Test 9
- ◯ Test 10

Point-of-Care Testing Survey

Build your own test - Question 6

	Probability of Correctly Diagnosed Positive	Probability of Correctly Diagnosed Negative	Cost Per Test	Time to Complete
Test 11	90 – 99%	99%	$50	25 min.
Test 12	90 – 99%	99%	$20	5 min.

Which test better suits your needs?

- ◯ Test 11
- ◯ Test 12

Build your own test - Question 7

	Probability of Correctly Diagnosed Positive	Probability of Correctly Diagnosed Negative	Cost Per Test	Time to Complete
Test 13	90 – 99%	90%	$20	25 min.
Test 14	90 – 99%	99%	$35	5 min.

Which test better suits your needs?

- ◯ Test 13
- ◯ Test 14

Point-of-Care Testing Survey

Build your own test - Question 8

	Probability of Correctly Diagnosed Positive	Probability of Correctly Diagnosed Negative	Cost Per Test	Time to Complete
Test 15	90 – 99%	95%	$35	15 min.
Test 16	90 – 99%	90%	$50	5 min.

Which test better suits your needs?

◯ Test 15

◯ Test 16

Point-of-Care Testing Survey

Build your own test - Question 9

	Probability of Correctly Diagnosed Positive	Probability of Correctly Diagnosed Negative	Cost Per Test	Time to Complete
Test 17	70 – 80%	99%	$50	5 min.
Test 18	90 – 99%	95%	$50	15 min.

Which test better suits your needs?

◯ Test 17

◯ Test 18

Point-of-Care Testing Survey

Build your own test - Question 10

	Probability of Correctly Diagnosed Positive	Probability of Correctly Diagnosed Negative	Cost Per Test	Time to Complete
Test 19	80 – 90%	99%	$50	25 min.
Test 20	90 – 99%	90%	$35	25 min.

Which test better suits your needs?

- ◯ Test 19
- ◯ Test 20

Point-of-Care Testing Survey

Build your own test - Question 11

	Probability of Correctly Diagnosed Positive	Probability of Correctly Diagnosed Negative	Cost Per Test	Time to Complete
Test 21	70 – 80%	99%	$50	5 min.
Test 22	90 – 99%	99%	$20	25 min.

Which test better suits your needs?

- ◯ Test 21
- ◯ Test 22

Point-of-Care Testing Survey

Build your own test - Question 12

	Probability of Correctly Diagnosed Positive	Probability of Correctly Diagnosed Negative	Cost Per Test	Time to Complete
Test 23	70 – 80%	95%	$50	15 min.
Test 24	90 – 99%	99%	$35	15 min.

Which test better suits your needs?

◯ Test 23

◯ Test 24

Point-of-Care Testing Survey

Build your own test - Question 13

	Probability of Correctly Diagnosed Positive	Probability of Correctly Diagnosed Negative	Cost Per Test	Time to Complete
Test 25	90 – 99%	95%	$35	5 min.
Test 26	70 – 80%	90%	$35	15 min.

Which test better suits your needs?

- ◯ Test 25
- ◯ Test 26

Point-of-Care Testing Survey

Build your own test - Question 14

	Probability of Correctly Diagnosed Positive	Probability of Correctly Diagnosed Negative	Cost Per Test	Time to Complete
Test 27	90 – 99%	90%	$20	15 min.
Test 28	90 – 99%	99%	$35	25 min.

Which test better suits your needs?

- ◯ Test 27
- ◯ Test 28

Point-of-Care Testing Survey

Build your own test - Question 15

	Probability of Correctly Diagnosed Positive	Probability of Correctly Diagnosed Negative	Cost Per Test	Time to Complete
Test 29	70 – 80%	99%	$50	5 min.
Test 30	90 – 99%	99%	$35	25 min.

Which test better suits your needs?

- ◯ Test 29
- ◯ Test 30

Point-of-Care Testing Survey

Build your own test - Question 16

	Probability of Correctly Diagnosed Positive	Probability of Correctly Diagnosed Negative	Cost Per Test	Time to Complete
Test 31	90 – 99%	95%	$35	15 min.
Test 32	70 – 80%	99%	$35	25 min.

Which test better suits your needs?

- ◯ Test 31
- ◯ Test 32

Point-of-Care Testing Survey

You have completed the survey.

Thank you very much for your time and input. Your contribution is vital for the success of new Point of Care Tests. If you have any questions, please email Dr. Charlotte Gaydos, Primary Investigator at cgaydos@jhmi.edu. For more information about our study, you can visit our site at http://www.hopkinsmedicine.org/Medicine/std/ .
